# Mechanistic Insights into the Allosteric Regulation of the Clr4 Protein Lysine Methyltransferase by Autoinhibition and Automethylation

**DOI:** 10.3390/ijms21228832

**Published:** 2020-11-22

**Authors:** Mina S. Khella, Alexander Bröhm, Sara Weirich, Albert Jeltsch

**Affiliations:** 1 Institute of Biochemistry and Technical Biochemistry, University of Stuttgart, Allmandring 31, 70569 Stuttgart, Germany; mina.saad@ibtb.uni-stuttgart.de (M.S.K.); alexander.broehm@ibtb.uni-stuttgart.de (A.B.); sara.weirich@ibtb.uni-stuttgart.de (S.W.); 2 Biochemistry Department, Faculty of Pharmacy, Ain Shams University, African Union Organization Street, Abbassia, Cairo 11566, Egypt

**Keywords:** protein methylation, protein methyltransferase, automethylation, enzyme regulation, enzyme kinetics

## Abstract

Clr4 is a histone H3 lysine 9 methyltransferase in *Schizosaccharomyces pombe* that is essential for heterochromatin formation. Previous biochemical and structural studies have shown that Clr4 is in an autoinhibited state in which an autoregulatory loop (ARL) blocks the active site. Automethylation of lysine residues in the ARL relieves autoinhibition. To investigate the mechanism of Clr4 regulation by autoinhibition and automethylation, we exchanged residues in the ARL by site-directed mutagenesis leading to stimulation or inhibition of automethylation and corresponding changes in Clr4 catalytic activity. Furthermore, we demonstrate that Clr4 prefers monomethylated (H3K9me1) over unmodified (H3K9me0) histone peptide substrates, similar to related human enzymes and, accordingly, H3K9me1 is more efficient in overcoming autoinhibition. Due to enzyme activation by automethylation, we observed a sigmoidal dependence of Clr4 activity on the AdoMet concentration, with stimulation at high AdoMet levels. In contrast, an automethylation-deficient mutant showed a hyperbolic Michaelis–Menten type relationship. These data suggest that automethylation of the ARL could act as a sensor for AdoMet levels in cells and regulate the generation and maintenance of heterochromatin accordingly. This process could connect epigenome modifications with the metabolic state of cells. As other human protein lysine methyltransferases (for example, PRC2) also use automethylation/autoinhibition mechanisms, our results may provide a model to describe their regulation as well.

## 1. Introduction

Post-translational modifications (PTMs) of proteins control many of their functional properties in a dynamic and reversible way [1,2]. Many PTMs have been discovered to date, including methylation, phosphorylation, acetylation, and glycosylation [1,3]. Protein lysine methylation on histones and non-histone proteins is one of the most important and abundant PTMs that can impact regulatory and functional mechanisms of the corresponding proteins, also linking the dysregulation of this modification to many diseases [2,4,5]. Histone PTMs control the differential organization of chromatin and the resulting effects on gene expression during development and disease [6,7].

The methylation of histone H3 at lysine 9 (H3K9) with consequent recruitment of reader proteins (Swi6, Chp2 and Chp1) in fission yeast and its homologs in humans (HP1α, HP1β and HP1γ) are crucial steps in the assembly and spreading of heterochromatin [8,9]. The fission yeast *Schizosaccharomyces pombe* (*S. pombe*) protein lysine methyltransferase (PKMT) Clr4, a homolog of the Drosophila Su(var)3-9 and the human SUV39H1 and SUV39H2 enzymes, introduces methylation of lysine 9 on histone H3 [10,11]. It is the only active H3K9-specific PKMT in this species. Due to the fact that the DNA methylation machinery is missing in *S. pombe*, Clr4 functions as a main enzyme for heterochromatin formation by coupling of H3K9me3 formation with RNAi [12] and via H3K9me2 generation it has important roles in RNAi and heritable silencing of transcription outside of heterochromatin [13]. Clr4 consists of 490 amino acids, comprising a chromodomain (CD) (8–60) at the N-terminus and a C-terminal Su(var)3-9, Enhancer-of-zeste and Trithorax (SET) catalytic domain (215–490). The CD binds methylated H3K9 [14] while the SET domain introduces the H3K9 methylation using *S*-adenosyl-l-methionine (AdoMet) as a methyl group donor [11,15]. A disordered region between the CD and SET domain binds to the nucleosome core also contributing to Clr4 activity [16].

In cells, Clr4 establishes a read–write mechanism through recognition of H3K9 methylation by its chromodomain followed by generation of more H3K9 methylation by the SET domain [14]. In conjunction with the interaction of Clr4 with Swi6, which also contains a chromodomain that binds methylated H3K9, this positive feedback loop causes spreading of the heterochromatic H3K9 methylation mark [17,18]. Similar to other PKMTs [19,20,21,22,23,24,25], Clr4 is inhibited by peptides and proteins containing a K-to-M mutation at the target site, H3K9M in this case, and expression of this histone mutant prevents heterochromatin spreading [26]. Moreover, Clr4 functions in complex with other proteins such as Cul4, Rik1, Raf1 and Raf2, forming the Clr4 multi-protein complex (CLRC) [27].

PKMT target residues are usually surrounded by a defined short sequence motif of amino acids, which are specifically recognized in a binding cleft located in the SET domain of the enzymes. The resulting substrate sequence specificities have been analyzed for many PKMTs using peptide and protein libraries as methylation substrates [28,29,30,31,32,33,34,35,36,37,38]. In a study investigating the Clr4 specificity sequence motif using peptide array methylation experiments, it was observed that Clr4 prefers R at the −1 position of its target site (related to the target lysine), and it shows some additional more relaxed preferences at the +1 (S > K, R >T) and +2 (T >> C > S) positions [39].

Very recently, it was shown that the Clr4 SET domain contains an autoregulatory loop (ARL) (amino acids 453–472) positioned between the SET and post-SET domains. A related loop is observed in other members of this PKMT family, but its sequence is not conserved [40]. This loop has been shown to have an autoinhibitory function, because it can bind to the enzyme active site, thereby blocking the H3 binding pocket and preventing catalytic activity [40]. Strikingly, in this conformation, K455 occupies a similar position to H3K9 in proximity to the sulfur atom of AdoMet. This residue (and also K472 in the ARL) were shown to be automethylated in cis, i.e., by the active center of the same enzyme molecule. Automethylation of these residues was shown to lead to a conformational switch of the ARL away from the active site, resulting in increased enzyme activity towards external substrates [40]. However, the mechanism by which the ARL modulates the enzyme activity and deposition of H3K9 methylation still needs more investigation.

In this study, we investigated autoinhibition and automethylation as one approach to regulate the activity of Clr4. Through site-directed mutagenesis of residues in the ARL, we stimulated or inhibited automethylation leading to corresponding changes in Clr4 catalytic activity. Furthermore, we demonstrate that Clr4 prefers monomethylated (H3K9me1) over unmodified (H3K9me0) histone peptide substrates similar to related human enzymes [33,41]. By determining the reaction rates of Clr4 at variable AdoMet concentrations, we observed a sigmoidal response with stimulation at increasing AdoMet concentration, while an ARL automethylation-deficient mutant (K455R/K472R) showed a canonical hyperbolic relationship. These data suggest that automethylation of the ARL could act as a sensor for AdoMet levels in cells and regulate the generation and maintenance of heterochromatin accordingly. As other human PKMTs (for example PRC2 [42,43]) also exhibit automethylation and autoinhibition, our results could provide a model to describe their regulatory mechanism as well.

## 2. Results

### 2.1. Site-Directed Mutagenesis, Clr4 Expression and Purification

**Figure 1:**
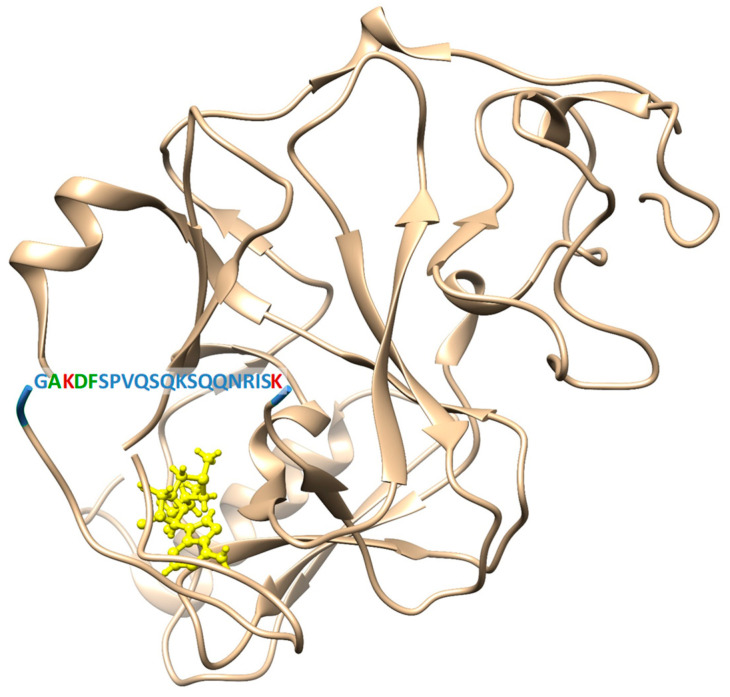
Crystal structure of the Su(var)3-9, Enhancer-of-zeste and Trithorax domain (SET domain) of Clr4 showing the sequence and approximate position of its autoregulatory loop (ARL), which is disordered in the structure (pdb 6BP4, [40]). The automethylated lysines K455 and K472 are marked in red, the amino acids surrounding K455 at −1, +1 and +2 positions which were mutated are marked in green.

In order to engineer Clr4 variants with altered automethylation activity, the sequence context of the automethylation sites in the ARL (AK_455_DF) (Figure 1) was compared with the Clr4 specificity profile, showing that they are not matching at −1, +1 and +2 positions (where the target lysine is annotated as zero). In our previous specificity analysis [39], Clr4 preferred R at the −1 position, where the ARL carries an A. Moreover, Clr4 showed some additional more relaxed preferences at the +1 (S > K, R > T) and +2 (T >> C > S) positions, where the ARL contains a DF sequence. Accordingly, we aimed to modify the ARL sequence to fit better to the Clr4 substrate sequence preferences, in order to increase the automethylation level and subsequently the methyltransferase activity. Therefore, A454 in the ARL was mutated to R (A454R mutant), D456 and F457 were mutated to S and T, respectively (D456S/F457T mutant), or all of the exchanges were combined in one mutant (A454R/D456S/F457T). Based on the fact that the histone mutant H3K9M inhibits Clr4 [26], another Clr4 variant was engineered, in which the target lysine in the ARL, K455, was mutated to M (K455M) to test if placing a methionine in the ARL could lead to a repression of Clr4 activity. Two more variants were created as automethylation-deficient mutants (Clr4 K455R and Clr4 K455R/K472R) where the automethylated lysine(s) in the ARL was substituted by arginine, which has similar physicochemical properties as lysine but cannot be automethylated. All variants were successfully created by site-directed mutagenesis using the Clr4 wild type (WT) gene as a template and confirmed by restriction digestion analysis and DNA sequencing. The SUMO- and His-tagged full-length Clr4 WT and variants were overexpressed in *Escherichia coli* cells and the proteins were purified by His-tag affinity chromatography followed by size exclusion chromatography. All protein constructs were successfully purified with good purity, as shown by loading equal protein amounts on an SDS-polyacrylamide gel (Supplementary Figure S1).

### 2.2. Effect of the Clr4 Mutations on Automethylation and Methyltransferase Activity

**Figure 2:**
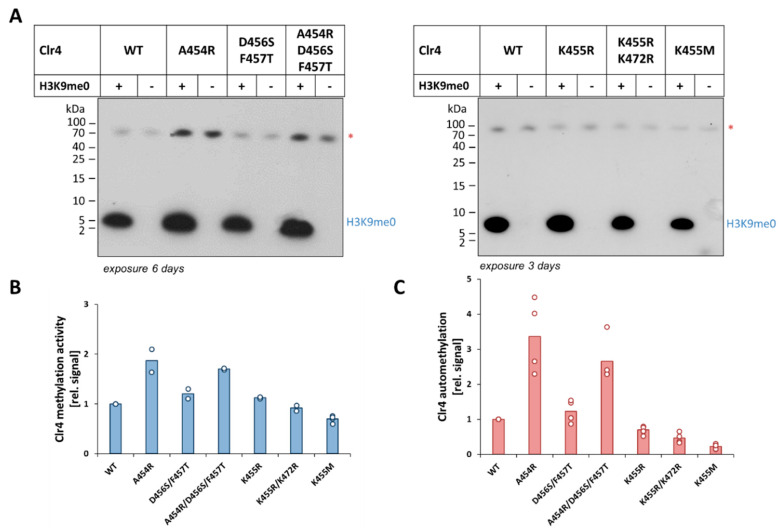
Methyltransferase activity and automethylation of different engineered Clr4 variants compared to wild type (WT). Exemplary autoradiographic gel images of in vitro methyltransferase assays with Clr4 WT and its mutants A454R, D456S/F457T, A454R/D456S/F457T, K455R, K455R/K472R and K455M (**A**). The red asterisks indicate the Clr4 automethylation signal. Quantitative analysis of two to four independent experiments revealed the histone peptide methylation activity (**B**) and automethylation levels (**C**) of all mutants, which are represented relative to WT enzyme. The mean values are indicated by the bars and the individual data points as circles. In panel (**C**), results obtained with and without peptide were combined. The *p*-values of the differences in automethylation and methyltransferase activities between WT and mutants are listed in Supplementary Table S1.

To test the effect of the different Clr4 ARL mutations on the level of automethylation and enzyme methyltransferase activity on histone peptide substrates, equal concentrations of the different enzymes were incubated with the unmodified H3K9 peptide (H3K9me0) in methylation buffer supplemented with radioactively labeled AdoMet as a cofactor. After the methylation reactions, the samples were separated by gel electrophoresis and the transfer of the radioactively labeled methyl groups from the cofactor to the respective target lysine residues in Clr4 and in the H3K9 peptide was visualized by autoradiography, analyzed quantitatively and normalized to WT (Figure 2 and Supplementary Table S1). Strikingly, our data revealed an about 3.4-fold stronger automethylation of the A454R mutant when compared to Clr4 WT (*p*-value = 0.012), indicating that the rational design of the ARL peptide sequence can increase automethylation. This increased automethylation was accompanied by an approximately two-fold higher methyltransferase activity on the H3K9 peptide (*p*-value = 0.004). In contrast, there was no significant change in automethylation and methyltransferase activity in the case of the D456S/F457T mutant, indicating that the effect of these residues is minor. This was also evident in the A454R/D456S/F457T triple mutant, which showed no significant difference from the A454R single mutant. The automethylation-deficient mutants K455R and K455R/K472R showed about 1.4- and 2.2-fold decreases in automethylation compared to the WT enzyme but, interestingly, this was not accompanied by a corresponding reduction in activity. In contrast, the K455M mutant, which acts as a suicidal enzyme inhibitor, caused a strong reduction in automethylation (about four-fold, *p*-value < 10^−90^) which was accompanied by a significant reduction in H3K9 peptide methyltransferase activity (30% less active, *p*-value = 0.0004).

### 2.3. Clr4 Prefers H3K9me1 over H3K9me0 as Substrate and Its Automethylation Is More Strongly Inhibited by H3K9me1 than by H3K9me0

**Figure 3:**
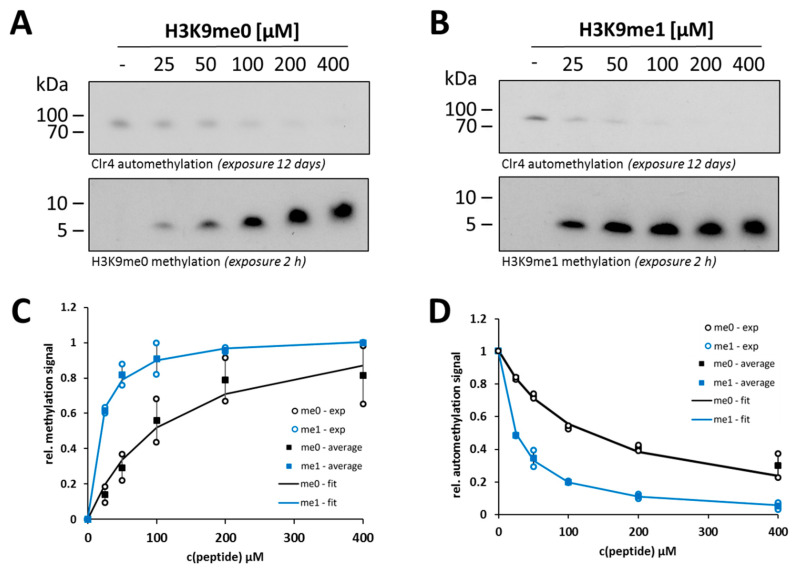
Methyltransferase activity and automethylation level of Clr4 WT at different concentrations of the H3K9me0 and H3K9me1 histone peptides. Exemplary autoradiographic gel images of in vitro methyltransferase assays with Clr4 WT using 25–400 µM H3K9me0 (**A**) or H3K9me1 (**B**) as substrate. (**C**) Michaelis–Menten model fit of the data for the histone peptide methylation activity observed in two independent repeats of the experiments shown in panel (**A**,**B**). All values are represented relative to the enzyme activity at 400 µM H3K9me1 peptide. (**D**) Analysis of the inhibition of automethylation by H3K9me0 and H3K9me1 peptides detected in two independent repeats of the experiments shown in panels (**A**,**B**). Data were fitted to an inhibition model and are represented relative to Clr4 enzyme automethylation in the absence of peptide. In panels (**C**,**D**), averages are shown as squares, error bars represent the SEM and individual data points are shown as circles. The kinetic parameters and inhibition constants are listed in Table 1.

Next, we were interested in comparing the steady-state kinetic parameters of H3K9me0 and H3K9me1 peptide methylation and their possible crosstalk with Clr4 automethylation. After confirming that similar concentrations of H3K9me0 or H3K9me1 peptides were used in the experiments (Supplementary Figure S2), the Clr4 WT enzyme was incubated with the H3K9me0 or H3K9me1 peptides in a concentration range of 25–400 µM in methylation buffer supplemented with radioactively labeled AdoMet as a cofactor. The methylated samples were separated by gel electrophoresis and the signals for automethylation and peptide methylation were detected by autoradiography using the same film exposure for both gels (Figure 3A,B). The quantified gel images for peptide methylation were used to derive the steady-state kinetic parameters of Clr4 by fitting to the Michaelis–Menten model (Figure 3C). Using the H3K9me0 peptide, we observed a K_m_-value of 118 µM for the WT Clr4 (Table 1), which is very similar to a previously published value (101 µM [44]). Our data demonstrate that Clr4 has stronger preference for the H3K9me1 peptide than for H3K9me0. This difference was mainly due to better binding of H3K9me1 peptide than H3K9me0 as indicated by an around seven-fold lower K_m_-value (Table 1).

For both peptides, Clr4 automethylation decreased with an increase in the peptide concentration, indicating competition between the ARL and external peptide substrate for access to the active site. The K_i_-values of the peptides on automethylation activity showed an around five-fold stronger inhibition of Clr4 automethylation by the H3K9me1 peptide than by H3K9me0 (Figure 3D and Table 1). This result confirms the stronger binding of H3K9me1 than H3K9me0 and quantitatively fits to the seven-fold reduced K_m_-value. These findings also indicate that monomethylated histone tail peptides can overcome the Clr4 autoinhibition better than the unmodified tails.

**Figure 4:**
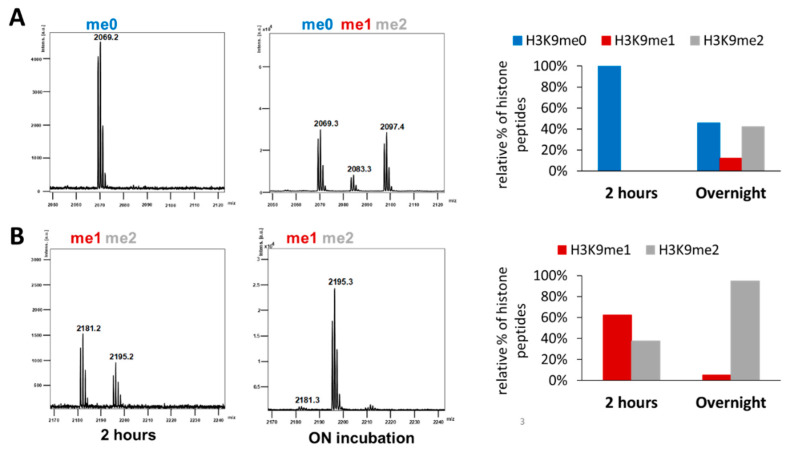
Detection of Clr4 activity on H3K9me0 and H3K9me1 histone peptides using MALDI-TOF mass spectrometry. Example of mass spectra for the different methylated histone peptides detected after 2 h or overnight (ON) methylation using Clr4 WT enzyme and 4.5 µM H3K9me0 (**A**) or H3K9me1 (**B**) in the presence of 1 mM unlabeled AdoMet. The relative fractions of histone peptides with different methylation states are indicated in the right panels. Mass spectra of the H3K9me0 and H3K9me1 histone peptides without enzyme incubation are shown in Supplementary Figure S3.

To confirm the preferential activity of Clr4 on H3K9me1, a second methyltransferase assay was conducted with unlabeled AdoMet using MALDI-TOF mass spectrometry for the detection of methylation activity. In agreement with the previous results, a higher preference for H3K9me1 over H3K9me0 as substrate was detected (Figure 4A,B, Supplementary Figure S3). After 2 h of incubation of WT Clr4 with equal concentrations of both peptides (H3K9me0 or H3K9me1) and the same concentration of unlabeled AdoMet, almost no methylation was detected with H3K9me0 (Figure 4A), while about 40% methylation was observed with H3K9me1 (Figure 4B). After overnight incubation, the samples showed 54% total methylation with the H3K9me0 substrate (12% H3K9me1 and 42% H3K9me2) (Figure 4A), but around 95% methylated H3K9me2 product was obtained with H3K9me1 (Figure 4B). The distribution of different methylated products observed after methylation of H3K9me0 also confirms the observation in the radioactive kinetics that H3K9me1 is the preferred substrate, because the absence of a transient accumulation of H3K9me1 demonstrates that the conversion of H3K9me1 to H3K9me2 is faster than the reaction converting H3K9me0 to H3K9me1.

### 2.4. Steady-State Kinetics with the Clr4 A454R Mutant Show Increased Automethylation and Less Autoinhibition

**Figure 5:**
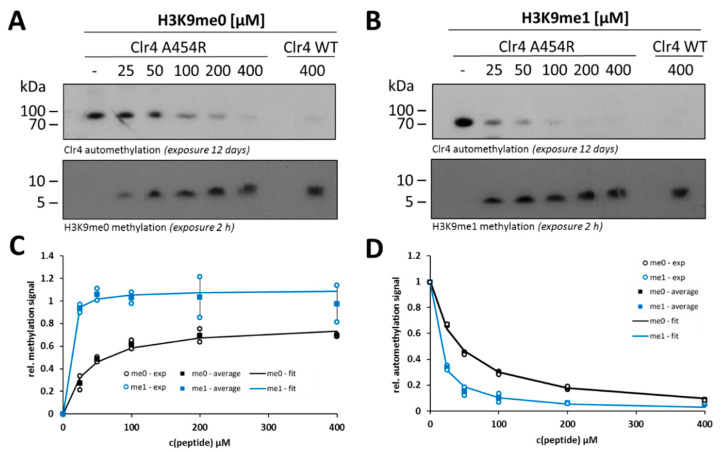
Methyltransferase activity and automethylation level of Clr4 A454R at different concentrations of H3K9me0 and H3K9me1 histone peptides. Autoradiography of the in vitro methyltransferase assay of A454R using 25–400 µM H3K9me0 (**A**) or H3K9me1 (**B**) as substrate. A reaction of WT enzyme incubated with 400 µM peptide was loaded on each gel as reference for the quantitative analysis. (**C**) Michaelis–Menten model fit of the data for the histone peptide methylation activity observed in two independent repeats of the experiments shown in panels (**A**,**B**). All values are represented relative to the WT enzyme activity at 400 µM H3K9me1 peptide. (**D**) Analysis of the inhibition of A454R automethylation by H3K9me0 and H3K9me1 peptides detected in two independent repeats of the experiments shown in panels (**A**,**B**). Data were fitted to an inhibition model and are represented relative to Clr4 enzyme automethylation in the absence of peptide. In panels (**C**,**D**), averages are shown as squares, error bars represent the SEM and individual data points are shown as circles. The kinetic parameters and inhibition constants are listed in Table 1.

Since the engineered Clr4 variant A454R showed the highest automethylation and methyltransferase activity, we were also interested in investigating its kinetic parameters and its automethylation in the presence of the two histone peptides at different concentrations, as analyzed before for Clr4 WT. Therefore, methylation reactions were conducted with radioactively labeled AdoMet using the same range of histone peptide concentrations as before (25–400 µM) and methylation signals were detected by autoradiography (Figure 5A,B). In all gels, one additional sample of Clr4 WT incubated with 400 µM histone peptide was loaded as a reference sample for further analysis. At low concentrations of both histone peptides (H3K9me0 and H3K9me1), the A454R mutant showed higher activity compared to the WT (Supplementary Figure S4), which is in agreement with our previous observation regarding this mutant activity. However, the impaired WT activity was largely rescued with increasing peptide concentrations, which illustrates the competitive inhibition between the histone peptides and the Clr4 ARL. The methylation reactions of H3K9me0 and H3K9me1 by A454R fitted to the Michaelis–Menten model showed similar trends as the WT (Figure 5C). For the A454R mutant, the K_m_-values for both substrates (H3K9me0 and H3K9me1) were about three-fold lower in comparison to WT (Table 1). This K_m_ difference explains the higher activity of the A454R mutant than WT, while the V_max_ difference was minor. Similar to with WT Clr4, the activity of A454R on the H3K9me1 peptide was higher than on H3K9me0, showing a seven-fold lower K_m_ (Figure 5C and Table 1). Regarding the inhibition of A454R automethylation by histone peptides, the data fitted to an inhibition model (Figure 5D) showed stronger inhibition by H3K9me1 than H3K9me0 similarly as seen with WT. The K_i_-values of H3K9me0 and H3K9me1 on automethylation of the A454R mutant were around three- to 2.5-fold lower than the corresponding values of Clr4 WT automethylation inhibition (Table 1).

### 2.5. Clr4 WT Reaction Rate Exhibited Sigmoidal Response towards Increasing AdoMet Concentrations

**Figure 6:**
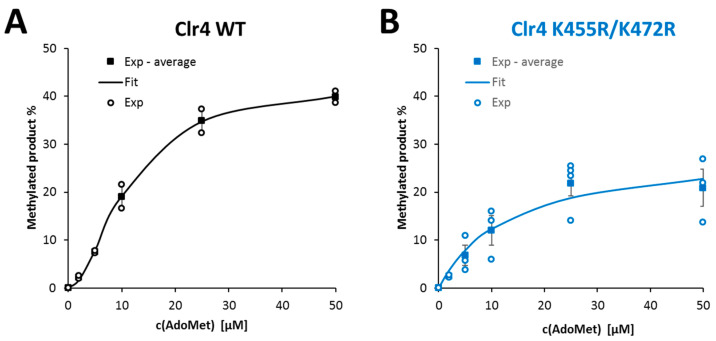
AdoMet dependence of the activity of Clr4 WT and the K455R/K472R mutant. Fractions of methylated products observed with increasing AdoMet concentrations (2–50 µM) for Clr4 WT (**A**) or the automethylation-deficient K455R/K472R mutant (**B**). Data were obtained from the analysis of corresponding relative peak intensities measured by MALDI-TOF mass spectrometry in two to four independent experiments (see Supplementary Figure S5). Averages are shown as squares, error bars represent the SEM and individual data points are shown as circles. The kinetic parameters obtained in the fitting are listed in Table 2.

To investigate the Clr4 AdoMet dependency and the role that could be played by ARL automethylation in sensing AdoMet concentrations, steady-state kinetics were conducted at variable AdoMet concentrations. Both Clr4 WT and the ARL automethylation-deficient K455R/K472R mutant were incubated with an equal concentration of histone peptide H3K9me1 in the presence of varying concentrations of unlabeled AdoMet (2–50 µM). Afterwards, the methylation products were detected by MALDI-TOF mass spectrometry (Supplementary Figure S5). The Clr4 reaction rates were derived from the analysis of relative peak intensities of the different methylated histone peptides (Figure 6A,B). Intriguingly, the Clr4 WT reaction rate showed a sigmoidal response towards increasing AdoMet concentration as described by a Hill model (Figure 6A, Table 2), while the automethylation-deficient mutant showed a regular hyperbolic shape, as described by the Michaelis–Menten model (Figure 6B, Table 2). These results were statistically validated and *t*-tests demonstrated that the Clr4 WT AdoMet concentration dependence cannot be convincingly fitted by a hyperbolic curve, but moving to a sigmoidal model did not improve the curve fit of the mutant data. Importantly, our data indicate that at low AdoMet concentrations (i.e., at very low levels of automethylation), both Clr4 WT and the K455R/K472R mutant showed similar activities. However, with increasing AdoMet concentrations, the activity of Clr4 WT was boosted and increased over the ARL automethylation-deficient mutant due to the sigmoidal response curve.

## 3. Discussion

**Figure 7:**
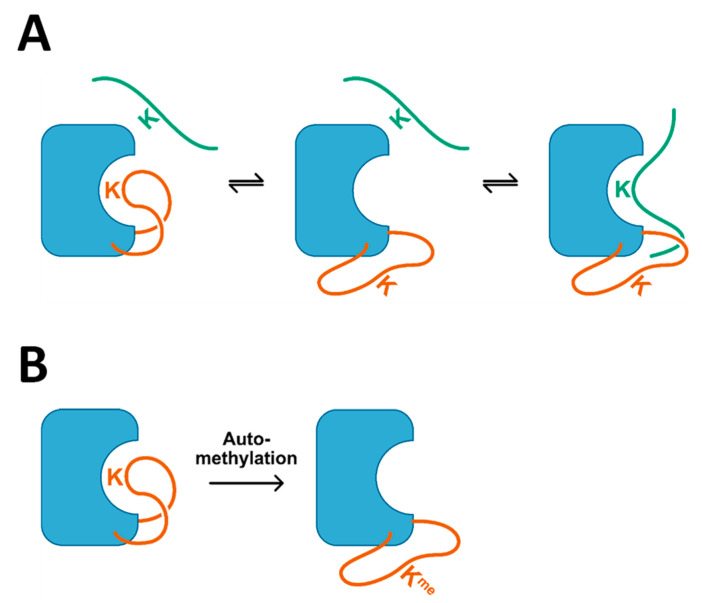
Model depicting the competition of the ARL (orange) and external peptide substrate (green) for access to the active site (**A**) and the shift of the conformation of the ARL towards the non-autoinhibited state by automethylation (**B**).

Previous data have shown Clr4 in an autoinhibited state, because its ARL binds to the active site cleft and thereby blocks binding of methylation external substrates [40]. Due to the positioning of lysine residues next to the active center, automethylation of Clr4 can occur at residues K455 and K472 in the ARL. Methylation of the ARL leads to its dissociation from the active site and relief of autoinhibition [40] (Figure 7). In this study, we used the modulation of the autoinhibition and automethylation through engineering of ARL mutants as an approach to fine-tune the Clr4 methyltransferase activity. Based on this, we report novel mechanistic insights into the automethylation-mediated allosteric enzyme regulation in terms of the histone peptide and AdoMet cofactor binding. The competition of the external peptide and ARL for binding to the active site is illustrated by our results obtained with the H3K9me0 and H3K9me1 peptides. H3K9me1 was found to bind more strongly to the enzyme and consequently it was more efficiently methylated and more efficient in overcoming the autoinhibition. The preference of Clr4 for H3K9me1 substrates observed here is in parallel with similar observations made with the related human enzymes SUV39H1 and SUV39H2 [33,41]. This result suggests that the Clr4 autoinhibition regulation may play a more prominent role in controlling the deposition of the first methyl group on H3K9, leading to the conversion of H3K9me0 into H3K9me1, than in the conversion of H3K9me1 into H3K9me2.

Regarding the Clr4 mutants, the designed A454R mutant showed an increased automethylation which was engineered by fitting the ARL sequence more to the Clr4 specificity sequence identified previously by our lab [39]. The increased automethylation shifts the conformation of the ARL towards the non-autoinhibited state and, consequently, enzyme activity was increased. In addition, a stronger inhibition of the A454R automethylation by the histone peptides in comparison with the WT was observed. This could be explained if the mutated ARL fits less well into the peptide-binding cleft, leading to a shift of the ARL conformation towards the non-autoinhibitory state. The strong automethylation of the A454R mutant indicates that once the ARL binds to the active center of Clr4, its methylation is more efficient due to the better sequence context of the target lysine. This means that the hyperactivity of the Clr4 A454R mutant is due to two effects, an increase in ARL automethylation and a less stable binding of the ARL to the peptide-binding cleft.

On the other side, automethylation of the K455R and K455R/K472R mutants was reduced, confirming the methylation of these residues. However, the residual automethylation persisting in the K455R/K472R mutant indicates that even more lysine residues in Clr4 can be automethylated. These additional automethylation sites must be outside of the ARL, because K464, the only remaining lysine residue in the ARL, has already been shown not to be methylated [40]. The catalytic activities of the K455R and K455R/K472R mutants on external substrates determined in radioactive methylation assays was not changed, because these assays were conducted at low AdoMet concentrations, such that automethylation levels were low even in the Clr4 WT and loss of the automethylation could not affect activity strongly. The K455M mutant containing an “in build” target lysine to methionine inhibitor revealed strongly reduced automethylation which was even lower than in the ARL automethylation-deficient mutants K455R and K455R/K472R. This effect is explained because K455 is no longer available for methylation and, additionally, because the strong binding of M455 into the active site blocks the methylation of other automethylation sites. At the same time, methylation of the external histone peptides was also reduced, resulting in lower methyltransferase activity of this mutant compared to WT. We believe that the approach used here for increasing or repressing of enzyme activity by modulation of the ARL can also be applied to other PKMTs with ARL.

The activity of chromatin-modifying enzymes is connected with the metabolic state of the cell by the concentrations of several important intermediates [45,46,47]: kinases utilize ATP as co-substrate, acetyltransferases acetyl-CoA, methyltransferase AdoMet, deacteylases of the sirtuin family NAD^+^, JmjC-domain lysine demethylases and TET methylcytosine dioxygenases molecular oxygen and 2-oxogluratare. In all these cases, fluctuating concentrations of the metabolites will affect chromatin signaling depending on the respective K_m_-value of the specific enzyme involved. Moreover, in many cases, different types of transferases can not only modify external substrates, but they can also modify themselves, leading to autophosphorylation, autoacetylation or automethylation. Automethylation of PKMTs and protein arginine methyltransferases (PRMTs), for example, has been described for many enzymes, including G9a [48], SUV39H2 [49], MLL1 [50], PRMD9 [51], SETD6 [52] and PRC2 [42,43] in the case of PKMTs and CARM1 [53], PRMT8 [54], PRMT6 [55] and PRMT7 [56], in the case of PRMTs. If automethylation is connected to the regulation of autoinhibition, as shown for Clr4 [40] but also other enzymes, for example, PRC2 [42,43], it provides a powerful tool to connect enzyme activity with the AdoMet concentration in the cell going beyond a simple K_m_-related modulation of enzyme activity. The reason for this is that automethylation and methylation of the external substrates both require AdoMet, suggesting that the enzyme can respond to increasing AdoMet concentrations with a stronger increase in activity than predicted by a simple Michaelis–Menten model. Indeed, our data demonstrate that the response curve of WT Clr4 to the AdoMet concentration is sigmoidal with stimulation at high AdoMet levels, while the mutant lacking automethylation in the ARL showed a normal hyperbolic response. The K_m_-value of Clr4 for AdoMet is within the range of intracellular AdoMet concentrations observed for yeast under different cultivation conditions (1–80 µM) [57,58], suggesting that the AdoMet concentration could directly regulate Clr4 activity through automethylation. As Clr4 is a key factor in the establishment and maintenance of heterochromatin in *S. pombe*, our data provide a connection between the metabolic state of *S. pombe* (here related to the available amounts of AdoMet) and epigenetic regulation. Our results suggest that Clr4 might act as sensor of the cellular AdoMet concentration and modulate heterochromatic H3K9me3 formation in response to fluctuating nutritional states or other signals that influence intracellular AdoMet concentrations. As exemplified by PRC2 [42,43], other human PKMTs also use automethylation/autoinhibition mechanisms, indicating that similar mechanisms may be common. Moreover, modulation of automethylation could have important roles in diseases. For example, one of the EZH2 cancer mutants is K510R, which affects the main site of PRC2 complex automethylation [43].

In conclusion, this study provides better understanding of the allosteric regulation of Clr4 by autoinhibition/automethylation, which can be considered as a model system for human PKMTs. It sheds light on potential effects of ARL mutations on enzyme activity, which could be biologically important for pathogenesis of disease-associated mutations. Moreover, it elucidates mechanisms by which the ARL can modulate Clr4 activity on histone tails depending on histone methylation state or AdoMet levels.

## 4. Materials and Methods

### 4.1. Site-Directed Mutagenesis, Protein Expression and Purification

All different Clr4 variants were created by site-directed mutagenesis using the megaprimer method [59] and confirmed by restriction digestion analysis and DNA sequencing. The plasmid encoding for SUMO- and His-tagged full-length Clr4 WT and different mutant variants were transformed into *E. coli* BL21-CodonPlus (DE3) cells (Novagen, Madison WI, USA) for bacterial expression. The bacterial cells were grown at 37 °C until they reached an optical density between 0.6 and 0.8. Afterwards, 0.2 mM isopropyl-β-d-thiogalactopyranoside (IPTG) was added to induce protein expression at 17 °C overnight. The next day, the cells were harvested by centrifugation at 4500 RPM for 20 min, followed by washing once with STE buffer (10 mM Tris-HCl pH 8.0, 1 mM EDTA and 100 mM NaCl) and collection of the cell pellets by centrifugation at 4500 RPM for 25 min.

For protein purification, the His-tag affinity chromatography method was used. In brief, the cell pellets were thawed on ice and resuspended in sonication buffer (30 mM KPI buffer pH 7.5, 500 mM KCl, 0.2 mM DTT, 1 mM EDTA, 10% glycerol, 20 mM imidazole) supplemented with protease inhibitor cocktail and the cells were disrupted by sonication. The lysed cells were centrifuged at 18,000 RPM for 90 min at 4 °C. The supernatant was passed through nickel–nitrilotriacetic acid (Ni-NTA; Genaxxon bioscience, Ulm, Germany) resin, which was pre-equilibrated with sonication buffer. Afterwards, the beads were washed twice with sonication buffer. The bound proteins were eluted with buffer containing 220 mM imidazole, 30 mM KPI, 500 mM KCl, 0.2 mM DTT, 10% glycerol. Purified proteins were analyzed by sodium dodecyl sulfate–polyacrylamide gel electrophoresis (SDS-PAGE) using 16% gels stained with colloidal Coomassie brilliant blue Fractions containing the protein were pooled and proceeded to gel filtration size exclusion chromatography. Size exclusion chromatography was performed using a Superdex 200 16/600 column (Merck, Darmstadt, Germany) which was connected to an NGC quest plus FPLC system (Bio-Rad, Hercules, CA, USA). The column was equilibrated in dialysis buffer (20 mM HEPES pH 7.2, 200 mM KCl, 0.2 mM DTT, 1 mM EDTA, 10% glycerol) and the protein sample, which was freshly eluted from the Ni-NTA column, was loaded at a flow rate of 1 mL/min using a 3 mL sample loop. The protein was eluted over at a constant flow rate of 1 mL/min. Fractions of 1 mL were collected, analyzed by SDS-PAGE and pooled according to purity. The Clr4 protein was eluted at a retention volume of 0.6 column volumes (70 mL), while His-tagged degradation products were eluted at 0.7 column volumes (80 mL). The protein solution was concentrated using Amicon Ultra-4 centrifugal filters (30 kDa cutoff), flash frozen in liquid N_2_ in aliquots and stored at −80 °C.

### 4.2. Peptide In Vitro Methylation Activity Assay

The H3K9me0 (1–19 aa) peptide was purchased from Intavis AG (Köln, Germany). Methylation reactions were performed by incubating the H3K9me0 peptide (2.4 µM) in methylation buffer (50 mM Tris pH 8.8, 10 mM MgCl_2_, 20 mM KCl, 1 mM DTT) supplemented with 0.76 µM radioactive labeled [methyl-3H]-AdoMet (PerkinElmer, Waltham, MA, USA) and 0.45 µM Clr4 WT or variants for 3 h at 30 °C. The reactions were stopped by the addition of Tricine-SDS-PAGE loading buffer followed by heating to 95 °C for 5 min. Afterwards, the samples were separated by Tricine-SDS-PAGE (4–16% gels), which was followed by the incubation of the gel in amplify NAMP100V (GE Healthcare) for 60 min on a shaker and drying of the gel for 2 h at 55 °C in a vacuum. The signals of the transferred radioactively labeled methyl groups were detected by autoradiography using a Hyperfilm^TM^ high-performance autoradiography film (Merck), which was placed on the dried gels at −80 °C in the dark. Films were developed with an Optimax Typ TR machine after different exposure times. For quantification, the signal intensities were measured with ImageJ (https://imagej.nih.gov/ij/) and analyzed with Microsoft Excel (Microsoft, Redmond, WA, USA).

### 4.3. Km Determination of H3K9me0 and H3K9me1 Substrates

The H3K9me0 (1–19) and H3K9me1-biotin (1–18) peptides were purchased from Intavis AG (Köln, Germany). The methylation reactions were performed using a peptide concentration range (25–400 µM) in methylation buffer (50 mM Tris pH 8.8, 10 mM MgCl_2_, 20 mM KCl, 1 mM DTT) supplemented with 0.76 µM radioactively labeled AdoMet (PerkinElmer) and 0.3 µM Clr4 WT or variants for 1 h at 30 °C. Afterwards, the peptide methylation experiments were performed as described above. Multiple turnover kinetics were determined using variable peptide concentrations (25–400 µM). Methylation levels were fitted by least squares fit with the Microsoft Excel Solver module under standard settings (GRL non-linear) to the Michaelis–Menten model (Equation (1)).

(1) $${v\;=\;v}_{\max}\frac{c_{s}}{c_{s}{\;+\;K}_{M}}$$

Equation (1): Michaelis–Menten model used to fit multiple turnover kinetics (v, reaction rate; c_S_, concentration of the substrate peptide; K_M_, Michaelis–Menten constant; v_max_, turnover number).

The inhibition of automethylation by different peptide substrates were globally fitted by least squares fit with the Microsoft Excel Solver module under standard settings (GRL non-linear) to Equation (2), which describes a simple inhibitor binding model.

(2) $${v\;=\;v}_{0}\frac{K_{i}}{c_{i}{\;+\;K}_{i}}$$

Equation (2): Inhibition model used to fit multiple turnover inhibition kinetics (v, reaction rate; v_0_, reaction rate in absence of inhibitor; c_i_, concentration of the inhibitor peptide; K_i_, inhibition constant).

### 4.4. Km Determination of AdoMet Using MALDI-TOF Mass Spectrometry

The methylation reactions were performed using the H3K9me1-biotin (1–18 aa) peptide (4.5 µM) in methylation buffer (50 mM Tris pH 8.8, 10 mM MgCl_2_, 20 mM KCl, 1 mM DTT) supplemented with unlabeled AdoMet (2–50 µM) (Sigma-Aldrich, St. Louis, MO, USA) and 0.9 µM Clr4 (WT or K455R/K472R mutant) for 3 h at 30 °C. The reactions were halted by the addition of 0.1% trifluoroacetic acid (TFA). All the samples were cleaned using C18 tips (Agilent Technologies, Santa Clara, CA, USA). The eluted samples were spotted on an anchor chip plate and allowed to dry completely. Later, 1 μL of HCCA matrix (0.7 mg/mL α-cyano-4 hydroxycinnamic acid dissolved in 85% acetonitrile, 0.1% TFA, 1 mM ammonium dihydrogen phosphate) was added to dried sample and allowed to dry again. After that, the dried spots on the anchor plate were analyzed using an Autoflex Speed MALDI-TOF mass spectrometer (Bruker-Daltonics, Billerica, MA, USA). The mass spectra were collected using the Flex control software (Bruker-Daltonics). For calibration, the peptide calibration standard (Bruker-Daltonics) with peptides ranging from 700 to 3200 Da was used. The collected spectra were then analyzed with Flex analysis software (Bruker Daltonics). The relative methylation levels were determined by using the corresponding peak intensities of the substrate peptide in different methylation states. Methylation levels were fitted by least squares fit with the Microsoft Excel Solver module under standard settings (GRL non-linear) to the Michaelis–Menten model (Equation (1)) or a Hill model (Equation (3)).

(3) $${v\;=\;v}_{\max}\frac{c_{s}{}^{n}}{c_{s}{}^{n}{\;+\;K}_{M}{}^{n}}$$

Equation (3): Hill model used to fit multiple turnover kinetics (v, reaction rate; c_S_, concentration of the substrate; K_M_, Michaelis–Menten constant; v_max_, turnover number; n, Hill coefficient).

## Figures and Tables

**Table 1:** Kinetic parameters of WT Clr4 and the A454R mutant obtained by steady-state methylation experiments conducted at variable peptide concentrations using radioactively labeled AdoMet as a cofactor. K_i_-values refer to the inhibition of automethylation by increasing concentrations of H3K9 peptide substrates. All data are given as mean ± SEM. The data and fits are shown in Figure 3 and Figure 5. Reaction conditions were c_enzyme_ = 0.3 µM, c_peptide_ = 0–400 µM, c_AdoMet_ = 0.76 µM. K_m_ refers to peptide binding at the specified AdoMet concentration. v_max_ values are given relative to WT activity with 400 µM H3K9me1 peptide.

Substrate	Clr4 WT			Clr4 A454R		
	K_m_ (µM)	rel. v_max_	K_i_ (µM)	K_m_ (µM)	rel. v_max_	K_i_ (µM)
**H3K9me0**	118.9 ± 9.4	1.13 ± 0.18	126.8 ± 7.9	37.2 ± 9.1	0.8 ± 0.05	43.3 ± 0.6
**H3K9me1**	16.38 ± 2.2	1.05 ± 0.02	24.6 ± 1.82	5.5 ± 1.6	1.14 ± 0.03	11.5 ± 1.6

**Table 2:** Kinetic parameters of WT Clr4 and the K455R/K472R mutant obtained by steady-state methylation experiments conducted at variable AdoMet concentrations using the H3K9me1 peptide substrate. All data are given as mean ± SEM. The data and fits are shown in Figure 6. Note that the mutant data were fitted to the Michaelis–Menten model corresponding to an n-value of 1. Reaction conditions were c_enzyme_ = 0.9 µM, c_peptide_ = 4.58 µM, c_AdoMet_ = 0–50 µM. K_m_ refers to AdoMet binding at the specified peptide concentration. v_max_ values are given relative to WT activity.

Substrate	Clr4 WT			Clr4 K455R/K472R		
	K_m_ (µM)	rel. v_max_	N	K_m_ (µM)	rel. v_max_	n
**AdoMet**	11.4 ± 1.5	1.0 ± 0.01	1.87 ± 0.19	13.6 ± 1.0	0.71 ± 0.08	1

## Supplementary Materials

The following are available online at https://www.mdpi.com/1422-0067/21/22/8832/s1.
