# Supplementary material for: Mechanistic Insights into the Allosteric Regulation of the Clr4 Protein Lysine Methyltransferase by Autoinhibition and Automethylation
Source: Int J Mol Sci. 2020 Nov 22;21(22):8832. doi: 10.3390/ijms21228832 (PMC7700585; doi:10.3390/ijms21228832)
Supplement: Supplementary file 1 [file ijms-21-08832-s001.pdf]

Supplementary materials

Mechanistic Insights into the Allosteric Regulation of the Clr4 Protein Lysine Methyltransferase by Autoinhibition and Automethylation

Mina S. Khella, Alexander Bröhm, Sara Weirich and Albert Jeltsch

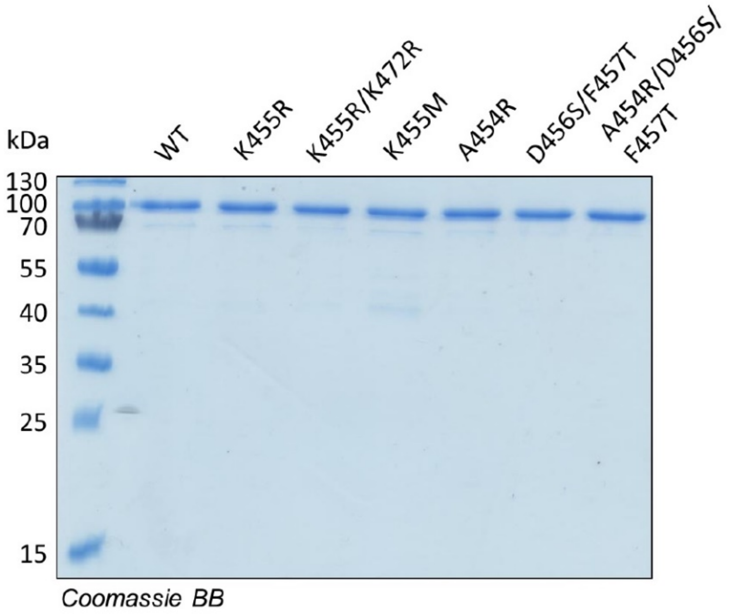

Figure S1. Coomassie BB stained SDS gel of the purified Clr4 WT and mutants showing high purity and comparable concentrations of the purified proteins.

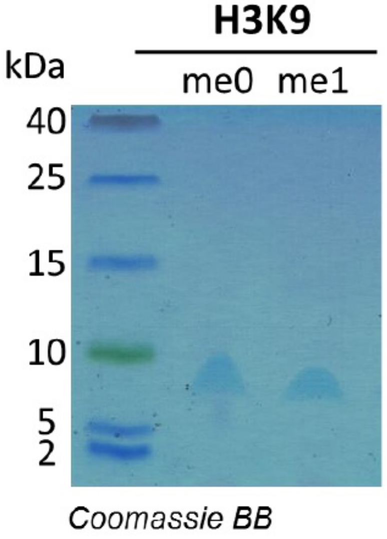

Figure S2. Coomassie BB stained Tricine SDS gel of histone peptides H3K9me0 and H3K9me1.

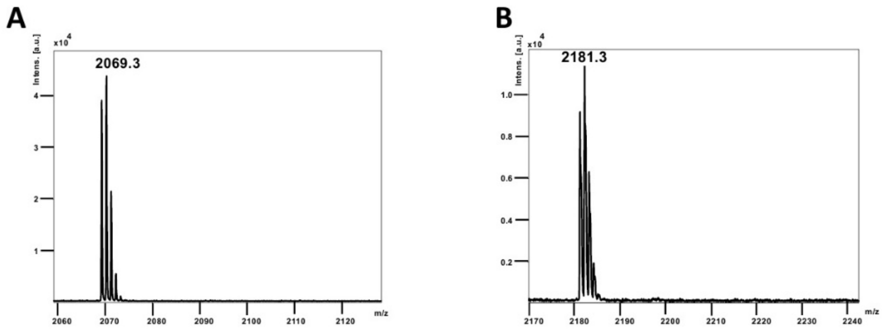

Figure S3. Mass spectra of the H3K9me0 (**A**) and H3K9me1 (**B**) histone peptides without enzyme incubation.

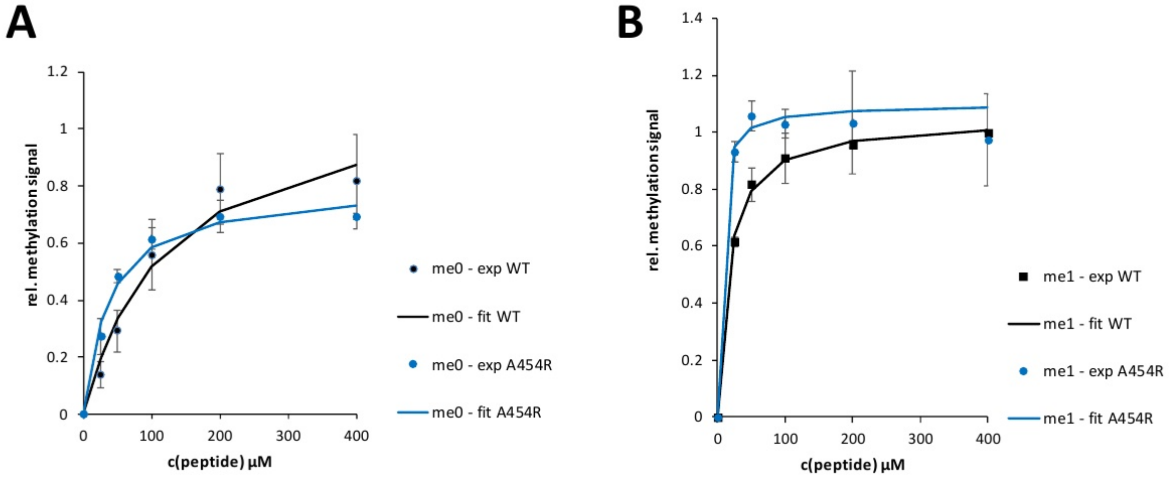

Figure S4. Comparison of the methyltransferase activity of Clr4 A454R and Clr4 WT at different concentrations of the H3K9me0 (**A**) or H3K9me1 peptide (**B**) as substrate.

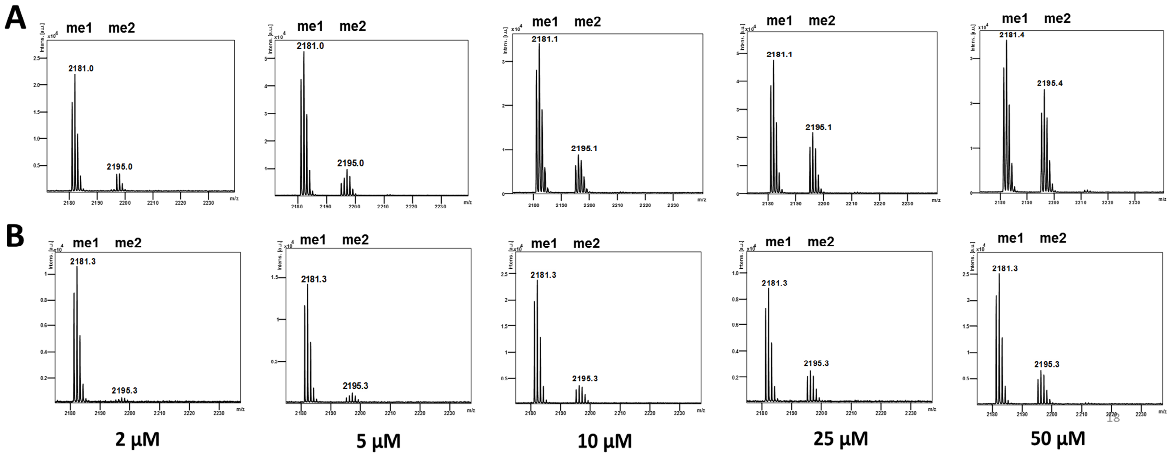

Figure S5. MALDI-TOF mass spectrometry methylation analysis of Clr4 WT (**A**) and the automethylation deficient mutant K455R/K472R (**B**) at different AdoMet concentrations (indicated below the panels). All samples were incubated for 3 h.

Table S1. *Z*-statistics derived *p*-values of differences in automethylation and methyltransferase activity levels of different Clr4 mutants shown in Figure 2 compared to wild type. All values are mean ± S.D. *P*-values < 0.05 were considered as significant. n.s.—not significant.

Clr4 Mutant	Automethylation Level (Relative Value)	*p*-Value	Methyltransferase Activity (Relative Value)	*p*-Value
A454R	3.4 ± 1.05	0.012	1.9 ± 0.33	0.004
D456S/F457T	1.2 ± 0.33	n.s.	1.2 ± 0.14	n.s.
A454R/D456S/F457T	2.7 ± 0.66	0.006	1.7 ± 0.02	$<10^{-90}$
K455R	0.7 ± 0.08	6.07×10^{-5}	1.1 ± 0.008	3.3×10^{-55}
K455R/K472R	0.46 ± 0.15	0.00015	0.91 ± 0.08	n.s.
K455M	0.23 ± 0.05	$<10^{-90}$	0.7 ± 0.09	0.0004
